# What Are Young Adults Saying About Mental Health? An Analysis of Internet Blogs

**DOI:** 10.2196/jmir.1868

**Published:** 2012-01-30

**Authors:** Madalyn A Marcus, Henny A Westra, John D Eastwood, Kirsten L Barnes, Mobilizing Minds Research Group

**Affiliations:** ^1^Department of PsychologyYork UniversityToronto, ONCanada; ^2^see acknowledgements

**Keywords:** Young adult, mental health, mental health services, life experiences, blogging, qualitative research

## Abstract

**Background:**

Despite the high prevalence of mental health concerns, few young adults access treatment. While much research has focused on understanding the barriers to service access, few studies have explored unbiased accounts of the experiences of young adults with mental health concerns. It is through hearing these experiences and gaining an in-depth understanding of what is being said by young adults that improvements can be made to interventions focused on increasing access to care.

**Objective:**

To move beyond past research by using an innovative qualitative research method of analyzing the blogs of young adults (18–25 years of age) with mental health concerns to understand their experiences.

**Methods:**

We used an enhanced Internet search vehicle, DEVONagent, to extract Internet blogs using primary keywords related to mental health. Blogs (N = 8) were selected based on age of authors (18–25 years), gender, relevance to mental health, and recency of the entries. Blogs excerpts were analyzed using a combination of grounded theory and consensual qualitative research methods.

**Results:**

Two core categories emerged from the qualitative analysis of the bloggers accounts: I am powerless (intrapersonal) and I am utterly alone (interpersonal). Overall, the young adult bloggers expressed significant feelings of powerlessness as a result of their mental health concerns and simultaneously felt a profound sense of loneliness, alienation, and lack of connection with others.

**Conclusions:**

The present study suggests that one reason young adults do not seek care might be that they view the mental health system negatively and feel disconnected from these services. To decrease young adults’ sense of powerlessness and isolation, efforts should focus on creating and developing resources and services that allow young adults to feel connected and empowered. Through an understanding of the experiences of young adults with mental health problems, and their experiences of and attitudes toward receiving care, we provide some recommendations for improving receptivity and knowledge of mental health care services.

## Introduction

Mental health problems are highly prevalent among young adults, with up to 25% experiencing a mental health problem in a given year [1-3]. Despite this high prevalence, young adults are particularly unlikely to seek help and, as a result, many do not receive adequate care [4-6]. Research has shown that stigma and negative beliefs about mental health care play a fundamental role in the decision to access and remain engaged in care [5,7-9]. Thus, to understand why young adults are particularly unlikely to access treatment, it is important to determine the specific beliefs and experiences of young adults with mental illness. Given the tendency of young adults to avoid seeking help, innovative ways of hearing from these hard-to-reach young adults must be explored. The Internet provides such a possibility.

### Going Online

The Internet has become a key space for health information sharing [10], with 78% of American young adults looking for health information online [11]. In fact, people are more likely to use the Internet to find health information than to go to a physician [12]. Moreover, the Internet is not solely a location to gather health information, as people frequently use online spaces to tell their stories and connect with others. This is especially the case with online journals or blogs, which are updateable public records of private thoughts [13]. Previously termed weblogs, blogs gained popularity in 1999 by providing individuals with the ability to write about their thoughts and feelings in a free, dedicated online space [14]. BlogPulse [15], a trend-discovery system for blogs, monitors over 155 million blogs with over 1 million updated in the last 24 hours. In 2010, 12% of Internet users wrote blogs, while 51% of Internet users read blogs [16]. Rates vary on the frequency of blog usage by young adults, with 18% to 39% of young adults reporting having written an online journal or blog [17,18]. Further the number of blogs and blog writers is growing exponentially [19,20]. Thus, the widespread use of the Internet has created a unique space for hearing from young people who may not have been consulted in previous health service utilization research.

Despite the large number of young adults using the Internet to share their experiences, few studies have examined these accounts to gain an increased understanding of the experiences of young adults with mental health problems. Rather, most Internet-based studies actively recruit participants using methods such as online surveys [21] or qualitative interviews [22-24]. Baker and Fortune [22] found through email-based semistructured interviews with young adults that an online self-harm or suicide community provides emotional support, valuable information, and friendship for individuals. While these studies provide vital information, they do so within the confines of a research study where participants are recruited, and thus their responses are influenced by participants’ awareness of the research context. This contextual knowledge introduces demand characteristics, which have been found to significantly influence participant responses [25].

### Present Study

The present study used an innovative approach to understand the overall experience and impact of living with a mental health problem among young adults. Namely, we conducted a qualitative grounded theory analysis of the blogs of young adults (18–25 years of age) who were specifically blogging about their experiences with mental health problems. As such, the present study allowed young adults’ experiences to be understood free from a research context, thereby providing a more unbiased account of the experience of living with a mental health problem.

## Methods

### Selection of Blogs

We used an enhanced search engine, DEVONagent (Mac OSx; DEVONtechnologies, LLC, Coeur d’Alene, ID, USA), to search online blogs with English text that included key words pertaining to mental health. Initial searches using keywords such as depression yielded a high volume of possible blogs. We subsequently iteratively refined searches by using keywords identified in pertinent blogs. Multiple searches were conducted using various combinations of the following key words: blog (diary, personal, personal experience), mental health (depressed, depression, anxious, anxiety, bipolar), and therapy (counseling, support, psychologist, psychiatrist, medication, Prozac, Celexa, etc). Later searches added the keywords youth and young adulthood. Subsequently, we manually searched the blogs from the initial searches. That is, we then reviewed each bloggers’ blog roll, which is a list of other blogs that the current blogger recommends. This allowed us to connect to other blogs that were also affiliated with mental health issues in order to identify other possible bloggers and major websites (eg, Anxiety Tribe, Depression Tribe, and PsychCentral) meeting criteria relevant to the study. Thus, we downloaded an exhaustive list of existing personal online and publicly available blogs on the theme of mental health issues and archived them onto a secure computer.

This generated a large database (approximately 3500 webpages), which was furthered refined in the following steps. First, we included only blogs from those 18 to 25 years of age. Blogs were excluded when no age was listed in the user profile or age could not be determined from the text of the blog. Second, we checked the blogs to ensure that the content of the blog pertained primarily to young adult discussion of mental health problems. Third, to ensure that selected blogs were recent and frequently updated, only blogs posted between August 2008 and February 2009 were included. Fourth, only blogs that were updated on a weekly or biweekly basis were retained. Fifth, we examined the frequency of viewing of the blog by others and removed blogs that had been viewed less than 200 times. At this stage in the data selection procedure, we had reduced our sample to 18 blogs, the majority of which were authored by women with mood disorders. To ensure more proportional representation of men and a greater variety of mental health concerns, we eliminated another 10 blogs from the sample that were authored by women and focused on mood disorders. Sixth, we retained excerpts only from the blogs pertaining to mental health for analysis and excluded irrelevant content (eg, events or discussion unrelated to mental health). In general, extraneous material not related to mental health was relatively rare, comprising less than 5% of the blog postings.

### Participants

In summary, our selection procedure resulted in a total of 8 blogs with the following characteristics: clearly authored by 18- to 25-year-olds; frequently updated and viewed; authored by 2 men and 6 women who had a variety of mental health problems; and written between August 2008 and February 2009.

**Table 1:** Sample characteristics of bloggers

Blogger	Gender	Age (years)	Location	Main mental health concern
1	Female	23	England	Bipolar disorder
2	Male	23	England	Social phobia
3	Male	22	Ireland	Bipolar disorder
4	Female	23	United States	Posttraumatic stress disorder/dissociative identity disorder
5	Female	21	England	Depression
6	Female	18	England	Depression/eating disorder
7	Female	22	Columbia	Depression
8	Female	18	England	Social anxiety

**Table 2:** Characteristics of blogs

Blogger	Total number of posts during time period	Number of text pages analyzed	Number of hits (at time of data collection)	Blog URL
1	130	30	500,000 hits since February 2007	http://thesecretlifeofamanicdepressive.wordpress.com/
2	51	29	1000 hits since August 2007	http://socialphobic.co.uk/
3	26	20	400 hits since May 2008	http://guyinterrupted.blogspot.com
4	73	12	231 hits since June 2008	http://crackersandjuice.blogspot.com/
5	59	29	7000 hits since May 2008	Not available
6	108	69	3000 hits since September 2008	http://blueskiesandgreengrass.wordpress.com/
7	50	42	36,000 hits since October 2006	http://crazyasuka.wordpress.com/
8	27	13	2,600 hits since September 2008	http://meryine.blogspot.com/

Table 1 presents characteristics of the final sample of 8 bloggers in this study. Table 2 presents information on the number of posts and hits for each blogger, the number of pages of text analyzed, and the blog URL. Across the 8 bloggers a total of 524 blog posts, comprising 244 pages of text, were reviewed and analyzed.

### Ethical Issues

This study was reviewed and approved by York University’s Institutional Review Board for research with human subjects. Only publicly accessible blogs were used in the present study. We excluded blogs requiring a username and password or registration form or fee for which the individual could clearly expect anonymity. The use of public rather than private information is consistent with recommendations for ethical conduct of research described by investigators conducting similar research and follows ethical conduct for Internet research in particular [26-28]. Further, the bloggers were individually contacted to obtain their consent for inclusion of quotes from their blogs and the URL for their blog in the presentation of the findings from the present study. Finally, all personally identifiable information was removed or changed.

### Data Analysis

We analyzed blog excerpts using a combination of grounded theory [29] and consensual qualitative research methods [30]. Qualitative methods offer a systematic, inductive way of investigating experience. Rather than being constrained by previously determined concepts, they allow researchers to examine experiences and phenomena as they naturally arise from the data. More specifically, grounded theory is a method of analysis that emphasizes the generation of theory that is grounded in the inductive investigation of participant experiences, in this case, blogger accounts of their experience of mental health problems [29,31,32].

In grounded theory, researchers first immerse themselves in the text by reading and rereading the text as a whole. After this the text is divided into units of meaning reflecting a complete thought, which stays as close to the language of participants as possible [32]. This is referred to as open coding. Similar-meaning units are then continuously grouped together as coding proceeds into larger-order categories, both within and across participants. Categories are continuously modified throughout the analysis by adding and subtracting meaning units. This is referred to as the constant comparative method [29], where data and the conceptualized categories are continuously compared. This process eventually results in a hierarchy of categories, with lower-order categories being subsumed by higher-order ones. Often, an overarching core category (or categories) emerges that organizes the relationship among all other categories and the data supporting them.

Grounded theory typically relies on a single researcher. In the present study, however, consensual qualitative methods supplemented the grounded theory analysis to integrate the perspectives of multiple researchers. In consensual qualitative research a team of researchers is used to make decisions on the data by consensus [30]. The team consisted of 3 researchers, with 2 coding each transcript, and the third serving as an auditor. In the present study, meaning units and categories were coded independently by 2 researchers and then a final designation of meaning units and categories was determined through discussion to achieve consensus. The 2 coders and the auditor came together to develop consensus on the meaning units and categorization of the data. A fourth person served as an additional auditor at the end of this process for consensus on the overall model.

To achieve diversity in perspective on the data, the 2 coders and the 2 auditors differed in level of experience in treating mental health problems, with 1 senior undergraduate psychology student, 1 advanced graduate student in clinical psychology, and 2 clinical psychologists with 10 and 16 years of experience in the field, respectively. It is important for qualitative researchers to be aware of subjective biases and, as much as possible, to put aside or bracket these biases, expectations, and hypotheses. As such, in an effort to mange their assumptions, the researchers wrote field notes and memos, bracketing and becoming aware of any biases or personal reactions to the data [33] in order to remain as objective as possible. Through writing memos and self-reflection, the researchers, at all points throughout the study design, data collection, and data analyses, attempted to exhibit reflexivity by constantly examining how the research process, including their potential biases, may affect the resulting research outcomes [34].

### Saturation

Saturation is the point at which the addition of new data does not add new information to the developing heuristic model. We considered the issue of saturation to be relevant both within and across bloggers. Within bloggers, meaning units were identified beginning with the most recent post and moving backward in time. During this process similar meaning units were placed together in categories. The coders independently identified the point at which no new categories were derived from reviewing additional blog posts. Coders then arrived at consensus through discussion, to identify the point of saturation across bloggers. In the present study, saturation across bloggers was achieved with 6 bloggers, since the addition of the last 2 bloggers did not yield any additional categories to the developing heuristic model.

## Results

**Figure 1:**
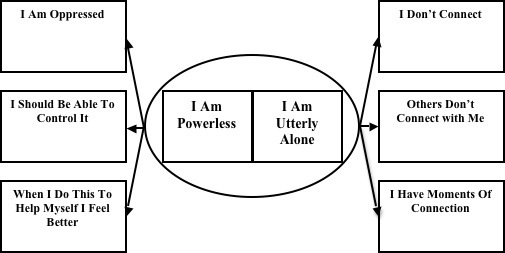
Emergent model of bloggers’ experience of a mental health concern.

Two core categories emerged from analysis of the bloggers accounts: *I am powerless* (intrapersonal) and *I am utterly alone* (interpersonal). Table 3 presents the number of bloggers who wrote content pertaining to a given theme and the number of instances of a given theme across all bloggers. The emergent model of bloggers’ experience of a mental health concern is depicted in Figure 1. The core categories and the themes (or categories and subcategories) they comprise are described further below.

**Table 3:** Model of bloggers’ experience of a mental health concern

Category				Number of bloggers	Number of meaning units across all bloggers
**Core category I: I am powerless**					
	I am oppressed				
		It dominates my life/stains everything		8	31
		It’s relentless		7	32
		It paralyzes me		8	19
		It’s exhausting		5	10
		It’s confusing/I don’t know what to do		8	24
		It makes me afraid		7	27
		It’s hopeless		8	19
		I think about suicide		5	11
	I should be able to control it			7	33
	When I do things to help myself I feel better				
		Self-care		7	29
		Taking medications		4	14
		Blogging		4	10
**Core category II: I am utterly alone**					
	I don’t connect				
		I hide my true feelings:		7	16
			Because I’m ashamed	5	13
			Because I’m concerned about others’ reactions	6	16
			Because I’m scared/unsure	3	16
		I’m all alone		5	13
		I lack the skill to connect		4	18
		I am a burden		4	13
		It’s my fault that I’m alone/I deserve it		7	28
	Others don’t connect with me				
		Others insist I have control when I don’t		8	30
		Mental health professionals are unresponsive			
			I have strong mixed feelings about medication	7	46
			I’m not getting enough care/I’m abandoned	6	42
			My care is inconsistent/disorganized	2	13
		Mental health professionals are unapproachable			
			They are unsupportive, hurtful, or intimidating	6	18
			They have their own agenda	5	10
		Previous lack of support contributed to my problem		3	18
	I have moments of connection:				
		Because of me		8	30
		Because of others		7	25
		Because of blogging			
			Blogging connects me to others who understand	6	14
			Blogging allows me to help others	3	8

### Core Category I: I am Powerless

Overall, young adult bloggers expressed significant feelings of powerlessness as a result of their mental health concerns. They described their mental health problems as an all-encompassing, highly destructive force, leaving virtually no aspect of their functioning and daily lives untouched. Young adults described feeling victimized and overpowered by their mental health problems, while simultaneously blaming themselves for not being able to control these experiences. They also articulated trying to engage in self-care activities, which provided some relief.

#### I am Oppressed

This category was defined by experiences of mental health problems as dominating, relentless, paralyzing, exhausting, confusing, frightening, and hopeless. At least 5 of the 8 bloggers contributed to each subcategory in the larger category of feeling oppressed, reflecting the commonality of these experiences across individuals with varied mental health problems.

##### It Dominates My Life/Stains Everything

All bloggers (8/8) contributed properties to this subcategory, describing mental health problems as permeating all aspects of their lives. This was often accompanied by strong feelings of frustration and resentment. For example, bloggers noted that

blogger 1 The biggest causalities have been my relationships with lovers, friends, family, my working life, my education, and my memory.

blogger 6 [My] moods go from low to high, and vice versa, continually...it’s ruining my life.

blogger 7 I’m not all about depression but it takes so much of me. It doesn’t define me but it stains everything I say and do.

##### It’s Relentless

The majority of the bloggers (7/8) described feeling chronically incapacitated by the relentlessness of their mental health symptoms, including the significant fear of symptom relapse. They reported experiencing disappointment and frustration at the recurring nature of their symptoms. Bloggers stated that

blogger 1 It’s 10 years of unchecked mental illness that has, over and over again, ruined whatever patchwork mockery of a life I had.

blogger 6 I don’t want to be like this. I thought I was recovering. I thought that this was over. But it really isn’t.

##### It Paralyzes Me

All bloggers (8/8) also described feeling restrained and controlled by their symptoms. For example, blogger 1 noted that

It’s like being locked in an iron maiden...[Depression] pushes one into the rut of not having the energy or inclination for very much at all, but feeling resentful and frustrated because of it.

And blogger 7 described that

Having depression is like having invisible cuffs. You don’t move and you shift between feeling like the idiot who can’t move although they have no restrains at all, and realizing the cuffs are indeed there but not being able to get rid of them.

##### It’s Exhausting

Relatedly, many of the bloggers (5/8) described the experience of having a mental health problem as exhausting. They spoke of the emotional and physical fatigue that was a constant aspect of their mental health problems. Bloggers also discussed their sleep difficulties:

blogger 7 [My mood] is stable, apart from irritability that is mostly due to sleep deprivation.

blogger 8 There is nothing worse than having a restless sleep when you need about 10 hours of it to keep you going and keep you sane.

##### It’s Confusing/I Don’t Know What to Do

All of the bloggers (8/8) described feeling perplexed by the symptoms themselves and about how to address them. For example:

blogger 4 I am stuck. I don’t know if I can get better...and I don’t seem to have the words to say what it is that I am going through. I cannot define my pain or explain it, I just have symptoms of it.

blogger 4 I don’t really know what I want or need, but something has to change.

##### It Makes Me Afraid

The majority of bloggers (7/8) also discussed how they feared the power their mental health problem had over them. As a result, they frequently expressed a need to be “on guard” and always aware of their symptoms. Blogger 1 noted, “It all happens so astonishingly quickly I have to be so aware,” and blogger 7 said:

Having atypical depression, my moods are relatively reactive. It becomes increasingly tiring to see yourself feeling better in response to something while being aware that as soon as that thing is gone, you’ll fall back. That leads to anxiety when something good is happening.

##### It’s Hopeless

All bloggers (8/8) expressed often profound pessimism and hopelessness about the future as a result of having a mental health problem. For example:

blogger 2 Every time I think about the future, I just can’t help but feel dread. The only comfort I have is knowing that at least I have the necessary physical ability to kill myself.

blogger 2 There’s really no hope for someone like me, I just cannot cope with the struggles and hard things life throws at you.

blogger 8 I feel like I’m being watched on stage. I’m wearing too much makeup, this isn’t who I am, I hate myself. I want to give up, I can’t stand this.

##### I Think About Suicide

Just over half of the bloggers (5/8) reported extreme hopelessness in the form of suicidal ideation and appeared to derive comfort from entertaining the possibility of suicide. Examples:

blogger 1 I still find the thought of suicide comforting.

blogger 2 I am pretty much resigned to ending my life now...I am too cowardly to slit my wrists...I’ll try harder to find a place to jump from...There is not one consistent mode of thinking or conscious thoughts about jumping off a bridge, it’s more of an overall feeling that comes in waves.

Bloggers also wrote about feeling their suicide would not affect anyone. For example, blogger 6 wrote

When I commit suicide people won’t remember me. The way I want it to be, because at the end of the day I’m not special to anyone, and the more people who can forget me the better.

#### I Should Be Able to Control It

Even though they felt highly victimized and experienced mental health problems as an oppressive force, the majority of the bloggers (7/8) believed they were to blame for their problems and should be able to control their symptoms. Bloggers blamed themselves for their current symptoms, as exemplified by blogger 7: “It is easier to blame the problem on something you can potentially control—yourself—than to blame it on external influences” and later wrote “You also wonder what the big deal is...You probably brought this on yourself” (blogger 7). Moreover, bloggers frequently expressed feeling guilty for not being able to control their symptoms. For example, blogger 2 wrote that “I’m honestly sick of my life, the constant guilt that I should be making more of the opportunities that I have been extremely lucky to have.”

#### When I Do Things to Help Myself I Feel Better

Despite feelings of profound powerlessness, the majority of the bloggers (7/8) described engaging in activities resulting in symptom improvement including self-care activities, taking medications, and blogging itself. When the young adults took active steps to decrease their symptoms, they experienced improvements physically, emotionally, and mentally.

##### Self-care

The majority of the bloggers (7/8) reported the use of helpful self-care activities. For instance, blogger 3 reported that

My mood is better today, less grim, more focused...I meditate to calm and center my mind, clear my head. Classical music (Chopin) plays in the background.

In addition to mental and physical activities, some bloggers wrote about changes in their thinking that were beginning to result in an improved mood. For example, blogger 8 said

I feel like a failure in a way, but the amount I’ve been through and personally achieved in my time off has been completely life changing. I’m a stronger, wiser, and more experienced person.

##### Taking Medications

Half of the bloggers (4/8) discussed the helpful aspects of taking medication for their mental health problems. For example, “The Xanax is helping me a lot...I’m looking forward to the future rather than being paralyzed by fear at the thought of it” (blogger 3).

##### Blogging

Interestingly, half of the bloggers (4/8) stated that they used blogging as a means of coping, and found that disclosing and discussing their experiences with mental health problems online served many functions, including self-reflection, self-help, and acquiring much needed support. For example:

blogger 1 The one thing that keeps me in treatment, and writing, is pure interest...It’s fascinating.

blogger 7 I write these blogs as a way to keep up with my own mood, to monitor my progress (or lack thereof)...To be honest, without the Internet this would be rather difficult, if not impossible.

blogger 8 I’d prefer to spend hours writing...My worries float away as every page is turned...it’s like hiding in my word world.

### Core Category II: I Am Utterly Alone

The young adult bloggers described a profound sense of loneliness, alienation, and lack of connection with others. Young adults reported hiding their feelings from others because they felt ashamed, were concerned about other’s reactions, felt that they were a burden, and were scared or unsure about receiving mental health care, which contributed to their feelings of loneliness. They also spoke of feeling alone and as if they did not fit into the world around them. The bloggers wrote about the strain their mental health problem put onto others, further contributing to feelings of isolation. Many of the bloggers in fact blamed themselves for being alone, as they felt they lacked the skills to connect with others and that they deserved this isolation. Young adults also indicated that others, including mental health professionals, did not connect with them, as they were unresponsive and unapproachable. Despite this prevalent experience of loneliness, they also wrote about moments of connection when they took active steps to engage with others. In particular, blogging they viewed blogging as a positive space for discussing their mental health concerns, which served to empower the young adults.

#### I Don’t Connect

##### I Hide My True Feelings

The majority of the bloggers (7/8) spoke about hiding their experiences of living with a mental health problem. For example, blogger 1 wrote:

In the light of someone speaking to me, I will smile and respond, but as soon as they turn their heads my face falls immediately.

Similarly, blogger 3 wrote:

I put on a cheerful face, an easy smile. But it’s all empty, it’s all a façade. I’m so exhausted all I do is take my pills, eat, and sleep. I’m barely functioning.

Young adults reported hiding their true feelings because of shame, concern about other’s reactions, and uncertainty about these feelings (which are described below).

###### Because I’m Ashamed

Bloggers (5/8) reported hiding their feelings because they were ashamed about experiencing a mental health problem and assumed that others would share their negative self-view. This experience of shame and inferiority was often accompanied by fear of asking for assistance. For example, blogger 6 noted:

The day I have to tell the truth. The day where everything is going to come out, like a big ball of wool unraveling. How I don’t eat, sleep and how I tried to throw myself off a bridge. Whilst making all this seem perfectly reasonable, and that I’m not really mental, honest. I know I need more help, I’m just scared of the consequences.

These bloggers perceived themselves as weak and requiring more help to get though daily life than the average person, which contributed to their experience of shame. For example:

blogger 1 I had no choice but to ask for help. It was shaming for me. I hated myself for it.

blogger 7 I feel ridiculous requesting a softer treatment [help], and the first thing that suffers is my pride. But if I don’t do something I’ll drop out for sure.

###### Because I’m Concerned About Others’ Reactions

Most bloggers (6/8) anticipated that others would not react supportively if they were honest about their experiences. For example, blogger 7 wrote

I almost never talk about the “real thing” outside this blog. People never react well even if they are well meant.

Many of the young adults minimized the severity of their symptoms when speaking with others in an effort to protect people around them. Blogger 2 spoke about being concerned about her impact on her therapist:

It makes her sad to hear what I have to say...I don’t really like it when she looks at me all concerned.

Young adult bloggers spoke frequently about forcing themselves to appear “normal” for fear that others’ negative reactions would make things worse for them. For example:

blogger 7 I do basic things trying to raise no suspicions [get myself food or put on clothes]...I don’t want them to know how wrong things really are, because then they’ll confront me, and I have no answers.

They also spoke of fear that others would reject them. For example:

blogger 4 [Wanting my therapist] to protect and comfort me is NOT ok, when you start wishing for them, people feel overwhelmed and suffocated, they perceive you as needy.

###### Because I’m Scared/Unsure

Some of the bloggers (3/8) wrote about feeling scared and ambivalent about receiving mental health care, which was another reason for hiding their feelings and not seeking help. For example:

blogger 2 I’ve emailed Samaritans...but they don’t really help much and I’m far too scared to phone them.

blogger 6 I want to find out about more services in [the city I live in] and talk to the university counseling service but I don’t have the confidence to.

##### I’m All Alone

Many of the bloggers (5/8) spoke about experiencing significant social challenges and feeling like outcasts from society. Examples of this profound sense of being alone and disconnected:

blogger 2 I don’t fit in there either. It seems like there’s no place for me in the world.

blogger 7 I feel disconnected from everything and everyone and I hate to pretend there’s a connection anyway (it requires too much energy and it’s futile).

This social isolation contributed to the negative experiences of living with a mental health problem.

##### I Lack the Skill to Connect

Repeatedly describing it as a “hard process,” half of the bloggers (4/8) reported that they lacked the skill to connect with others. As blogger 2 reported, “It’s hard being alone all the time. What little social skills I had have atrophied.” The lack of connection with others contributed to their beliefs that they were unworthy of friendship and were incapable of forming social connections. Textbox 1 shows an excerpt from a poem blogger 8 wrote about this experience:

**Excerpt From a Poem Posted by Blogger 8**

I know I’m a terrible friend,

It’s a place I lack experience.

Always afraid to speak unless spoken to,

Wanting to run and hide.

Friends come along, get bored and move on.

For I’ve been hurt so many times,

Again I’m used.

Again I’m empty

Again I’m alone.

##### I Am a Burden

Half of the bloggers (4/8) wrote about how the strain their mental health problem put on family and friends further contributed to feelings of isolation. For example:

blogger 1 It’s exhausting being depressed for the both of us [boyfriend and me].

blogger 2 My parents are already ashamed of me, the stupid overgrown child who can’t handle life.

Consequently, young adults reported feeling guilty because of the negative impact their symptoms had on those around them.

##### It’s My Fault That I’m Alone/I Deserve It

The majority of the bloggers (7/8) blamed themselves for their experience of loneliness. That is, they felt they somehow deserved their mental health symptoms and the resulting social isolation. For example, “I feel embarrassed and moronic, why should I inflict my worthless, helpless self on anyone?” (blogger 8), and “Everybody else manages fine, why not me?” (blogger 8).

#### Others Don’t Connect With Me

Young adults described experiencing alienation through others’ invalidating insistence that people with mental health problems should and can exert control over their experiences. Moreover, young adults also described finding mental health professionals unresponsive and unapproachable. The bloggers also attributed their current problems, at least in part, to previous experiences of lack of support and caring from others.

##### Others Insist I Have Control When I Don’t

Consistently across all bloggers (8/8) was the expectation from others that their mental health problem should be easily manageable and, consequently, they experienced others as dismissive and offering simplistic advice. This is distinct from the intrapersonal feeling, discussed above, that one lacks control, because here bloggers are emphasizing the expectations of others. Textbox 2 shows a sample excerpt describing the frustration the bloggers feel as a result of this expectation.

Furthermore, the young adults wrote about being expected to just “get over it” and continue with regular activities of daily living. For example:

blogger 8 I’m constantly expected to perform tasks I struggle with. My family somehow forget that I’ve rarely ever been able to make phone calls, speak to people.

blogger 7 The attitude of my family is very telling. It is acceptable for me to feel awful because of something physical, as it is my body and I can’t control it, right? But, mentally? *gasp* No way! I cannot feel awful, I have to be strong. Doing otherwise implies weakness and a failure as a person however physical this really feels.

**Excerpt From Blogger 1 Describing the Frustration With Others’ Expectation That Mental Health Problems Are Easily Manageable**

Some of you reading this will of course take the angle that I can’t feel that bad because I’m sitting upright and writing this. Ah well. I feel guilty that I’m not cycling through the streets with hot cross buns in my basket like Mary Poppins. If someone were to whisper in my other ear that exercise cures depression I would probably punch them with the hand that I’m not using to chain smoke. I like to exercise, it makes me feel good but right now I simply don’t have the energy. I would just veer straight into a wall. I do all that jazz you’re supposed to do to “help yourself.” I’d like someone to explain how positive thought helps when your mind specifically boycotts positive thoughts from entering the building.

##### Mental Health Professionals Are Unresponsive

###### I Have Strong Mixed Feelings About Medication

The majority of the bloggers (7/8) expressed substantive ambivalence toward medication as a result of prior negative experiences, such as side effects. For example

blogger 1 I hate, hate, hate taking medication. I honestly cannot decide if it is for the best or not.

blogger 3 Managing my illness my way didn’t work...[Taking lithium] is the sentence I’ve been handed down. Let me think of it as freedom, and not my doom.

###### I’m Not Getting Enough Care/I’m Abandoned

The majority of young adult bloggers (6/8) reported feeling abandoned by the mental health care system, which resulted in a further sense of isolation and decline in functioning. Examples of this experience:

blogger 2 They can’t help me except by cramming pills down my throat but that doesn’t fix anything.

blogger 6 Tell him [the general practitioner] about everything...and I got a referral back to the primary care mental health team. I wasn’t exactly pleased, because I knew that I needed a lot more than a waiting list and no therapy.

###### My Care is Inconsistent/Disorganized

Some of the bloggers (2/8) described their mental health care as incoherent, confusing, and unpredictable. Blogger 6 stated that “I’m just wondering if my referral will ever go through.”

##### Mental Health Professionals Are Unapproachable

###### They Are Unsupportive/Hurtful/Intimidating

Many of the young adult bloggers (6/8) reported experiencing their psychiatrist or therapist, in addition to being neglectful, as intimidating, hurtful, and unsupportive. Blogger 7 expressed discontent toward her physician:

I’m tired of the psychiatrist and her dull look who just sits and writes things on her chart attempting one thing after the other like it was nothing for me to take one failure after another “hey! it didn’t work! Let’s try something else!

Compounding this experience was the bloggers’ described resistance to the therapist due to their lack of support. For example:

blogger 2 I get scared he’s [the psychiatrist] going to get angry at what I say, especially after he smashed his fist on the table after I deigned to ask him about antianxiety medication.

blogger 4 The therapist really does not like my eating disorder. It seems to be the one thing she doesn’t have a lot of compassion for and wants me to just do what I always do.

###### They Have Their Own Agenda

In addition to experiencing the mental health professionals as unsupportive, the bloggers (5/8) wrote about the professionals’ lack of regard for the young adult’s opinion. For example, “The appointment was fairly useless, apart from making me feel really crappy...The problem is that X knows very little about my current issues, purely because I didn’t get to air them” (blogger 6).

##### Previous Lack of Support Contributed to My Problem

A few of the bloggers (3/8) identified that lack of support from others was a contributor to their current problems. For example:

blogger 2 I think the enormous lack of social contact and life experience is as much to blame for my low mood [as]...any physiological cause of depression.

blogger 6 I never got praised, I was never allowed to play with messy things, and if I cried I got told I was making a fool of myself, so that plus bullying throughout high school has [led] me to this point.

blogger 8 I think that the years of bad experiences I had at school has etched this “fear” into me, where I perhaps have an underlying issue that causes this “mental block” or disability.

#### I Have Moments of Connection

Young adult bloggers described feeling moments of connection as a result of their own initiative and the initiative of others.

##### Moments of Connection Because of Me

All bloggers (8/8) reported that when they initiated a conversation with friends, family, or a mental health professional they felt less lonely, which resulted in an improvement in their mood. The following are sample properties from the bloggers describing the positive impact they experienced through initiating connections with others:

blogger 3 Thing are beginning to look much better...I met our friends...all in all it was wildly entertaining, and I got to socialize with my boss [and others] so it was all rather relaxed and thoroughly enjoyable. Very beneficial to my mood state.

blogger 2 I’ve been feeling extremely hopeless and suicidal again. It often seems to disappear as soon as I get the chance to talk to someone about it and I feel like I’m being overly dramatic by bringing it up but this time I was determined to speak the truth about it.

##### Moments of Connection Because of Others

The majority of bloggers (7/8) also wrote about the positive impact of others reaching out to them. For example, “I felt very accepted and cared about by my family which is something I am unfamiliar with” (blogger 4).

##### Moments of Connection Because of Blogging

Blogging also provided a space in which young adults could connect with others in a safe and supportive environment.

###### Blogging Connects Me to Others Who Understand

Many of the bloggers (6/8) spoke about the supportive nature of blogging. That is, blogging allowed for the formation of interpersonal relationships and a sense of community, which often did not exist in the nonvirtual world. For example, blogger 7 wrote about the connections made through blogging (Textbox 3)

**Blogger 7’s Description of Connections Made Through Blogging**

For months I’ve been blogging about it; not thinking much about it, I’ve ended up knowing a lot of people who suffer of mental disorders. In the circle of depressive blogs, you find people who understand what you’re going through. To be honest, without the Internet this would be rather difficult, if not impossible. When I see myself going through the worst, I think “what a pathetic, weak, (insert several other horrendous adjectives) person” despite my clinical knowledge of it. But when I read other bloggers going through the same, I want to hug them and for a second I see my own depressed self as someone worthy of the same support. You could say other blogs act as a mirror that is not being distorted by my own self-judgment.

###### Blogging Allows Me to Help Others

Subsequently, some of the bloggers (3/8) felt that their writing and detailed accounts of mental health problems serve as a helpful resource tool for other people with mental health problems. For example:

blogger 1 One of the reasons I keep this blog is to give a different impression of what someone with a severe mental illness is like, to show that people like me are just ordinary people with mental illnesses and individual personalities.

blogger 2 Hopefully, whatever I leave on this blog will serve as a cautionary tale to anyone going through the same experiences and hopefully they will change before it’s too late.

## Discussion

The present study used qualitative methods to analyze unsolicited blog entries of young adults to learn about their experiences of living with a mental health problem and to explore their attitudes and beliefs about mental health and treatment. In summary, the young adults described very significant suffering and impairment resulting from their mental health problems. Their experience of living with a mental health problem can be summarized into two core categories: (1) I am powerless, and (2) I am utterly alone.

In terms of the core category *I am p* *owerless* or without agency, young adults experienced their mental health problem as oppressive and overwhelming, yet they criticized themselves for not being able to control their difficulties and felt that they ought to be able to cope better. When seeking supports they experienced the mental health system as disempowering and controlling, and yet when they were able to engage in self-care they reported feeling better. In terms of isolation, young adults reported not connecting with others and that others did not connect with them. They noted feelings of profound isolation arising from shame, fear, guilt, and lack of ability to connect with others. Further compounding this isolation was that bloggers experienced others as failing to understand and failing to appreciate or show compassion for the difficulties the bloggers faced. Moreover, mental health professionals were experienced as unresponsive and unapproachable. Young adults perceived this lack of support and connection as contributing to their problems. Moments of connection, when they did occur, were experienced as restorative and as a source of hope.

Taken as a whole, the present results are consistent with the findings of previous research on young adults’ mental health attitudes [35,36] and mental heath literacy [37,38]. For example, previous studies found that young adults, in contrast to other age groups, do not believe that it will be helpful to seek care for their mental health concerns [39]. This may be exacerbated when young adults have past negative experiences of seeking professional help, as described by some of the bloggers in the present study [35]. Moreover, previous research has shown that young adults are significantly less likely to report interest in receiving professional care, such as with primary care doctors and medications [36,40,41]. Rather, young adults prefer to handle their concerns on their own or with the support of friends and family [9,37,38,42].

Importantly, the present findings expand what we know from earlier research. For example, the profound feelings of powerlessness, struggle, loneliness, and isolation that these young adult bloggers write about has not been highlighted or deduced from existing quantitative studies [9,35]. The present study uncovered the experiential sense of living with a significant mental health problem—not just the young adults’ attitudes, beliefs, or preferences [35,43,44]. In particular, while loneliness has been explored in past research [45,46], the profound feeling of aloneness described by the bloggers has not been highlighted in previous studies and perhaps should be a key goal of interventions or approaches to helping young adults with mental health problems. Mental health professionals, family, friends, and other allies should approach young adults with a firm sense of validation, understanding, empathy, and compassion.

Previous research has consistently found and documented that young adults prefer to handle mental health problems on their own and eschew the mental health system [37,38,47]. However, this previous work has not highlighted or investigated why this might be. Rather, the focus has typically been on developing ways to educate young adults about mental health treatment and convince them of the need for care and the benefits of care [48,49] in the absence of understanding why they might prefer to go it alone.

The present study suggests that one reason might be that young adults view the mental health system negatively and, for some, their experience has been consistent with this view. These findings strongly imply that the mental health care system should invest in efforts to educate others (lay people and mental health professionals alike) to create more welcoming, supportive environments that also facilitate choice in care. For example, not everyone wants medication [43,47,50] and it is imperative that there be engaged choice in treatment options, as this leads to higher treatment adherence and improved outcomes [51,52]. Efforts should also focus on creating and developing resources that allow young adults to feel connected (eg, blogging and informal supports). The creation of more accepting environments and attitudes will in turn facilitate greater self-acceptance among young adults, as an antidote to substantive self-criticism and self-blame. Indeed, the creation of more validating, empowering, and socially integrating mental health care treatments may be a more effective way to increase help seeking among young adults, compared with efforts aimed at improving mental health literacy.

Results of the present analysis also suggest that the act of blogging had several potentially therapeutic outcomes for the bloggers. First, the bloggers indicated that writing blogs was a way of expressing their inner emotions and difficulties. In this way writing provided a vehicle for the bloggers to reflect on their experiences in written form and gain understanding and sense of mastery over their problems. This finding is consistent with past research exploring the potential benefits of writing [53]. Many studies have shown that therapeutic writing can actually decrease symptoms of mental health concerns [53,54] and improve physical health [55]. In fact, therapeutic writing is sometimes used in psychotherapy contexts to assist in improving insight and mental health [56-58]. Second, the bloggers spoke about how blogging was an important communication medium for them, especially when they experienced such a profound sense of disconnection from the rest of their lives. In this way, blogging can be useful for self-expression, sharing, and decreasing a sense of loneliness [59,60].

These two key reasons for blogging, therapeutic writing and social connections, are also reflected in Technorati’s state of the blogosphere [61] study. This study of blogging more generally identified three distinct reasons for blogging: (1) self-expression, (2) sharing expertise and experiences, and (3) making money or doing business. The first two reasons are the most prominent motivators for blogging. Similarly, Nardi and colleagues [62] suggested five key motivations for blogging: (1) to chronicle life in order to share with others, (2) to express opinions and commentary in order to influence others, (3) to seek feedback and the views of others (eg, form participation), (4) to clarify or articulate one’s own thinking through the act of writing, and (5) to express deep emotions and release tension. The benefits of using this vehicle, blogging, for self-expression and connections is worthy of further exploration in future studies. These factors could be particularly important to people, such as the young adult bloggers in the present study, who feel isolated and disconnected in their offline lives.

### Limitations and Future Directions

The present study had a few important limitations. In particular, not all young adults with mental health problems choose to write blogs about their experiences. Further, the present sample was likely representative of higher symptom severity, and the bloggers were more likely treatment refractory, than the general population of young adult with mental health problems. As such, the participants were not representative of all young adults with mental health concerns. Future studies should be conducted with a broader range of young adults such as those who do not blog about their experiences and who have less severe problems or who may be at earlier stages of problem development or treatment.

The young adults examined in the present study have previously been difficult to hear from, and thus their voices have not shaped mental health treatment policies and programs. Moreover, the in-depth exploration of this particular group of young adults has generated important ideas for reforming mental health services for all young adults—namely, the creation of more validating, empowering, and socially integrating mental health care treatments. We suggest that future research ought to systematically examine what it is that young adults want to know about mental health problems and what treatment environments would be most conducive to young adults’ seeking care. In our view such research programs would contribute greatly to solving the problem of poor utilization of mental health care services among young adults.

### Conclusions

Even though young adults are very likely to have mental health difficulties, they are highly unlikely to seek or obtain mental health services, compared with other age demographics [63,64]. Thus, understanding the experiences of young adults with mental health problems, and their experiences of and attitudes toward receiving care, is critical to informing interventions and outreach efforts to better address these problems. The present study is one attempt to more fully understand these experiences, and the findings have several implications for meeting the needs of young adults with mental health problems. Through this infodemiology study, analysis of the bloggers’ accounts can be used to inform improvements to public health for young adults experiencing mental health concerns [65]. The findings of the present study also support the value of Internet research for gaining insight and understanding into the lives of individuals with mental health illnesses [66]. Individuals with severe mental health problems are a very important population, as they are among the most distressed and costly to the mental health care system. As such, it is recommended that researchers continue to use Internet communication vehicles to reach out to this underserved and undertreated population for improving receptivity and knowledge of mental health care services, as well as building trust and awareness among the community at large for understanding the complexity of mental health illnesses.
